# Crystal structures of the solvent-free and ethanol disolvate forms of 4,4′-(diazenediyl)bis(2,3,5,6-tetrafluorobenzoic acid) exemplifying self-stabilized azo­benzene *cis*-configurations

**DOI:** 10.1107/S2056989018012781

**Published:** 2018-09-25

**Authors:** Igor Elkin, Thierry Maris, Patrice Hildgen, Christopher J. Barrett

**Affiliations:** a Department of Chemistry, McGill University, Montreal, Quebec, H3A 0B8, Canada; b Department of Chemistry, Université de Montréal, Montreal, Quebec, H3C 3J7, Canada; c Faculty of Pharmacy, Université de Montréal, Montreal, Quebec, H3C 3J7, Canada

**Keywords:** crystal structure, self-stabilized *cis*-configuration, 4,4′-(diazenediyl)bis(2,3,5,6-tetrafluorobenzoic acid), substituted azo­benzene moiety

## Abstract

*cis*-4,4′-(Diazenediyl)bis(2,3,5,6-tetrafluorobenzoic acid), C_14_H_2_F_8_N_2_O_4_, and its ethanol disolvate, C_14_H_2_F_8_N_2_O_4_·2C_2_H_5_OH, represent new examples of self-stabilized *cis*-configured azo­benzenes obtained by a common crystallization procedure at room temperature under normal laboratory lighting conditions. The target structure constitutes of two 2,3,5,6-tetra­fluoro­benzoic acid residues linked to each other by a *cis*-configured azo group and was confirmed for two isolated specimens extracted from the same sample, corresponding to a solvent-free form and an ethanol disolvate. In the solvent-free form, the mol­ecule is characterized by rotational symmetry around a twofold rotation axis bis­ecting its central N=N bond while this symmetry is not present in the solvated form. The values of the inclination angles of the terminal carboxyl groups towards the corresponding benzene rings vary from 5.2 (4) to 45.7 (2)°, depending on the crystal composition. In the unsolvated form, the mol­ecules are linked through identical hydrogen bonds with a classical *R* ^2^_2_(8) graph-set ring motif of carb­oxy­lic acids, by generating supra­molecular chains running approximately parallel to [101]. The presence of ethanol in the solvated form also leads to changes in the short-contact pattern to produce both the *R* ^4^_4_(12) ring and open-chain motifs with alternating alcohol and di­carb­oxy­lic acid mol­ecules.

## Abstract 2

The synthesis and the crystal structure of *cis*-2,3,5,6,2′,3′,5′,6′-octa­fluoro-4,4′-azinodi­benzoic acid with and without residual ethanol are reported.

## Chemical context   

The parent structure of azo­benzene and its numerous differently substituted derivatives is comprised of two aromatic benzene rings separated by an azo group. One of the most intriguing properties of these artificial mol­ecules is their capability to shape reversibly the configuration of the azo group from the linear *trans* form, usually more stable, to the bent *cis* form, in the presence of an appropriate light irradiation, *e.g*. lasers or LEDs. Such controlled *trans*-*cis* inter­conversions at the mol­ecular scale, typically performed on the microsecond time inter­val or faster, have been amplified successfully to a macroscopic material photomechanical response, suggesting a highly promising route toward creating and applying diverse photoresponsive systems (Mahimwalla *et al.*, 2012 ▸; Bushuyev *et al.*, 2018 ▸). In this context, azo­benzenes, capable of adopting long-term stabilized *cis*-forms, represent an important tool for studying the *trans*–*cis* isomerization mechanisms, as well as for tuning the photomechanical properties. Particular attention has therefore been paid to polyfluorinated azo­benzene derivatives employed as components of various photoresponsive homo- and heteromolecular crystals (Bushuyev *et al.*, 2013 ▸, 2014 ▸, 2016*a* ▸,*b* ▸).
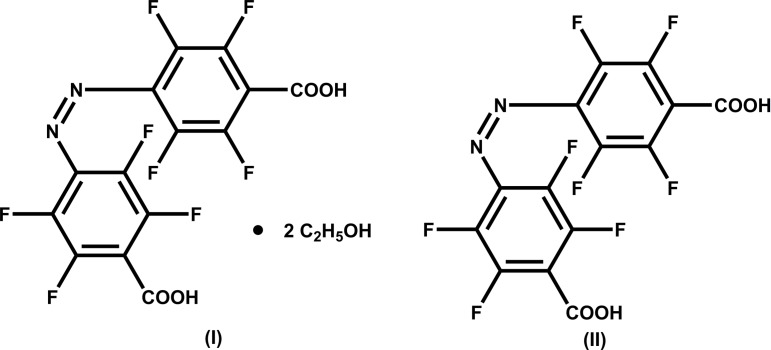


In the present study, we report the crystal structures of 4,4′-(diazenediyl)bis(2,3,5,6-tetrafluorobenzoic acid) with (I) and without residual ethanol (II), both adopting the *cis* configuration during a common crystallization procedure from the same solution in ethanol at room temperature under normal laboratory lighting conditions.

## Structural commentary   

**Figure 1:**
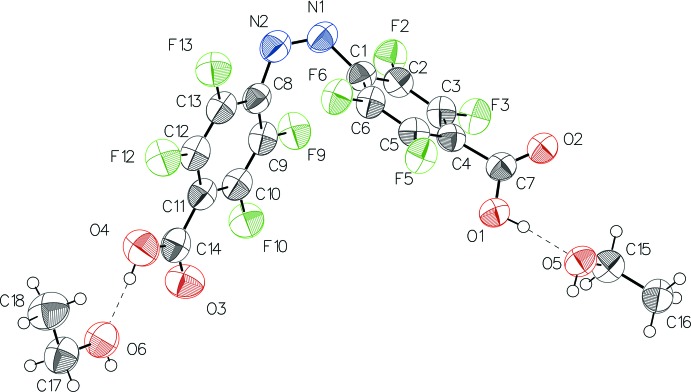
The mol­ecular structure of (I) showing the atom labelling and displacement ellipsoids drawn at the 50% probability level. H atoms are drawn as spheres of arbitrary radius, and hydrogen bonds are shown as dashed lines.

The mol­ecular structure of the title compound with (I) and without residual ethanol solvent mol­ecules (II), Figs. 1 ▸ and 2 ▸, respectively, is constituted of two 2,3,5,6-tetra­fluoro­benzoic acid residues linked to each other by a *cis*-configured azo group. In the solvent-free form (II), the mol­ecule is characterized by rotational symmetry around a twofold rotation axis bis­ecting its central N=N bond while this symmetry is not present in the solvated form (I).

In both types of crystal, the mol­ecular configurations are characterized by similar bond lengths and angles, which are in the expected ranges and are consistent with known data for *cis*-configured 2,3,5,6,2′,3′,5′,6′-octa­fluoro­azo­benzene moieties (Bushuyev *et al.*, 2013 ▸, 2014 ▸, 2016*c* ▸). Depending on the type of crystal, the two carboxyl groups are inclined differently to the planes of the corresponding benzene rings to which they are attached. In the ethanol disolvate form (I), the angles of inclination for groups O4—C14—O3 and O1—C7—O2 are 5.2 (4) and 45.7 (3)°, respectively, while in the solvent-free form (II), the value for O1—C7—O2 is 40.4 (3)°. The torsion angles between the central N=N bond and the two attached benzene C atoms are nearly the same in the two mol­ecules, *viz*. −9.8 (9)° for C1—N1=N2—C8 in (I), and −9.4 (4)° for C1—N1=N1^i^—C1^i^ [i) −*x* + 1, *y*, −*z* + 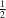] in (II).

## Supra­molecular features   

**Figure 3:**
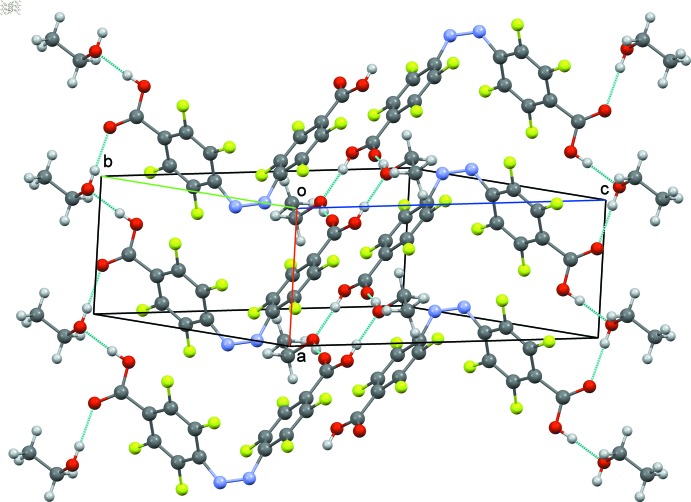
Partial view of the packing of (I), showing the hydrogen-bonding inter­actions (dotted lines). Hanging hydrogen bonds were omitted for clarity.

**Figure 4:**
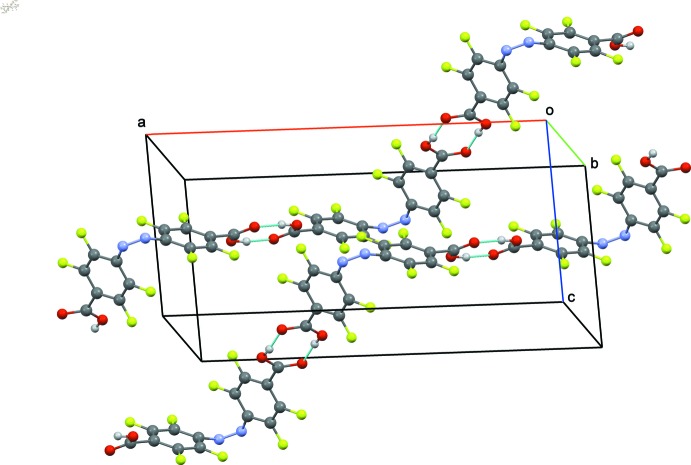
Partial view of the packing showing two hydrogen-bonded chains in (II). Hydrogen bonds are shown as dotted lines and hanging hydrogen bonds were omitted for clarity.

The inclusion of ethanol mol­ecules in the crystal composition renders different the patterns of inter­actions through hydrogen bonds for the forms (I) and (II) (Tables 1 ▸ and 2 ▸, respectively). For the solvated structure (I), the hydrogen bonds between the alternating hy­droxy groups of residual ethanol and the carboxyl groups of the title mol­ecule are arranged in two different ways, by forming either 12-membered rings involving four mol­ecules (two mol­ecules of each component), according to graph-set descriptor 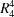
(12) (Etter *et al.*, 1990 ▸), or an open-chain pattern extending parallel to [100] (Fig. 3 ▸). As a result of such a configuration of short contacts, the whole supra­molecular scaffold is stabilized by mol­ecules belonging to adjacent parallel (011) layers stacked along [100]. For the unsolvated structure (II), the mol­ecules are organized in (10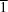) layers composed of identical corrugated chains running along [101]. In this case, the supra­molecular integrity is maintained primarily by the classical 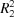
(8) ring motif of hydrogen bonds between the closest carboxyl groups (Fig. 4 ▸). A very similar motif was also observed in a solvent-free crystal of another 4,4′-dicarboxyl-substituted azo­benzene, *i.e. trans*-4,4′-(diazenediyl)di­­benzoic acid (Yu & Liu, 2009 ▸).

## Database Survey   

A search in the Cambridge Structural Database (Version 5.39 with one update; Groom *et al.*, 2016 ▸) returned 32 entries for different 4,4′-(diazenediyl)bis(2,3,5,6-tetrafluorobenzoic acid) derivatives and their co-crystals with other compounds. This includes the crystal characterization of pure *trans*-2,3,5,6,2′,3′,5′,6′-octa­fluoro­azinodi­benzene (Saccone *et al.*, 2014 ▸), *cis*- (Bushuyev *et al.*, 2016*c* ▸) and *trans*-2,3,4,5,6,2′,3′,4′,5′,6′-deca­fluoro­azo­benzene (Chinnakali *et al.*, 1993 ▸; Bushuyev *et al.*, 2016*c* ▸), as well as of co-crystals of the latter with *trans*-stilbene (Bruce *et al.*, 1987 ▸) and *trans*-azomesitylene (Bruce & Tiekink, 1989 ▸). The structures of other entries found by the search are also limited to the 4,4′-dihalide derivatives, *i.e*. to 4,4′-di­bromo- and 4,4′-di­iodo- ones, in their *cis* and *trans* configurations (Bushuyev *et al.*, 2013 ▸), as well as to their co-crystals with *cis*- and *trans*-1,2- bis­(4-pyrid­yl)ethyl­ene and *trans*-4,4′-azo­pyridine (Bushuyev *et al.*, 2014 ▸), with 4,4′-bi­pyridine, 4-methoxyl-4′-stilbazole and di­methyl­sulfoxide (Saccone *et al.*, 2014 ▸), with 1,4-di­aza­bicyclo­[2.2.2]octane, di­thiane, 4-vinyl­pyridine (Bushuyev *et al.*, 2016*c* ▸) and with *trans*-4,4′-di­cyano­azo­benzene, *trans*-4,4′-di­nitro­azo­benzene, *trans*-4,4′-azo­pyridine, 4-cyano-4′-pentyl­biphenyl and 1,10-phenanthroline (Bushuyev *et al.*, 2016*b* ▸).

## Synthesis and crystallization   

The title compound was synthesized according to a modified general protocol for obtaining symmetrically substituted azo­benzenes from the corresponding initial anilines (Clarke, 1971 ▸). Briefly, 3 g (0.014 mol) of 4-amino-2,3,5,6-tetra­fluoro­benzoic acid was neutralized in 60 ml of water by NaOH solution and adjusted to pH ≃ 8.5–9.0, and added dropwise to 100 ml of the commercial bleach solution Clorox^TM^ (The Clorox Company of Canada Ltd., ON, Canada), preliminary cooled to 273–278 K in an ice bath. The mixture was allowed to reach room temperature with overnight stirring. The resulting red-coloured solution was first treated with 80 ml of acetone and stirred for 1 h, to neutralize the excess of NaOCl, and then with aqueous HCl to pH 1.0 to give a pink sediment. After filtering and drying overnight at room temperature, the solid crude product was purified by extraction with ethanol followed by filtering. The final removal of solvent under reduced pressure gave 1.2 g of the target product with the yield of 40.4%. The structure and purity of the desired product were confirmed by LC–MS analysis performed on an Agilent Technologies 1260 Infinity LC–MS spectrometer (Santa Clara, CA, US) in ESI positive and negative modes. Separation was performed with an Agilent Poroshell 120 EC–C18 2.7 mm column, using as eluent the 0–100% gradient of solvent mixtures *A* and *B* [where *A*: water–aceto­nitrile (95%_vol_–5%_vol_) and acetic acid (0.1%_vol_); *B*: aceto­nitrile (99.9%_vol_) and acetic acid (0.1%_vol_)] under the following conditions: a capillary voltage of ESI source of 3000 V; a vaporizer temperature of 442 K, a nebulization pressure of 55 psig, a dry gas temperature of 571 K and a gas flow of 5 l min^−1^. Crystals of the title compound were obtained by vapor diffusion at room temperature using an ethanol solution in a small open vial placed in a sealed larger vessel filled with hexane. The ethanol solvate crystals were in the form of small yellow platelets while the unsolvated form crystallized as large orange plates.

## Refinement   

Crystal data, data collection and structure refinement details are summarized in Table 3 ▸. The H atoms of the hy­droxy and carboxyl groups in (I) were first positioned from Fourier synthesis and refined leveraging a riding model with *U*_iso_(H) set to 1.5 times *U*_eq_(O). All other H atoms of (I) were treated by using appropriate constraints. For (II), all the H atoms, including those belonging to the carboxyl group, were positioned from the difference synthesis and fully refined. For (I), non-merohedral twinning was found using the TwinRotMat Routine in *PLATON* (Spek, 2009 ▸). The twin law matrix was found to be (1 0 0, −0.621 − 1 0, −0.951 0 − 1). Processing the data as a two-component specimen with *SAINT* (Bruker, 2013 ▸) and *TWINABS* (Bruker, 2013 ▸) did not lead to an improvement in the refinement. Therefore, the initial data set was kept with the refinement performed using the HKLF5 file as generated with *PLATON*. The final BASF parameter indication the ratio of the two crystal domains was 0.646 (10).

## Supplementary Material

Crystal structure: contains datablock(s) I, II, global. DOI: 10.1107/S2056989018012781/wm5460sup1.cif

Structure factors: contains datablock(s) I. DOI: 10.1107/S2056989018012781/wm5460Isup2.hkl

Structure factors: contains datablock(s) II. DOI: 10.1107/S2056989018012781/wm5460IIsup3.hkl

CCDC references: 1866891, 1866890

Additional supporting information:  crystallographic information; 3D view; checkCIF report

## Figures and Tables

**Figure 2:**
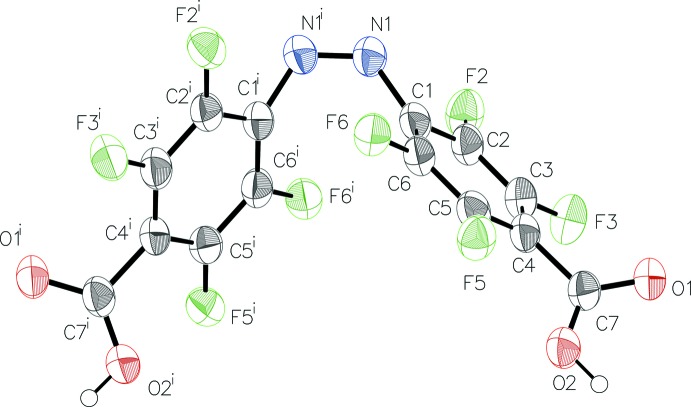
The mol­ecular structure of (II) showing the atom labelling and displacement ellipsoids drawn at the 50% probability level. H atoms are drawn as spheres of arbitrary radius. [Symmetry code: (i) −*x* + 1, *y*, −*z* + 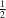].

**Table 1:** Hydrogen-bond geometry (Å, °) for (I)

*D*—H⋯*A*	*D*—H	H⋯*A*	*D*⋯*A*	*D*—H⋯*A*
O1—H1⋯O5	0.84	1.72	2.556 (5)	171.9
O4—H4⋯O6	0.84	1.74	2.579 (7)	175.7
O6—H6⋯O3^i^	0.84	1.93	2.751 (7)	164
O5—H5⋯O2^ii^	0.84	1.93	2.771 (6)	175.2

Symmetry codes: (i) 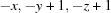; (ii) 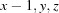.

**Table 2:** Hydrogen-bond geometry (Å, °) for (II)

*D*—H⋯*A*	*D*—H	H⋯*A*	*D*⋯*A*	*D*—H⋯*A*
O2—H2⋯O1^i^	0.90 (5)	1.71 (5)	2.607 (2)	173 (5)

Symmetry code: (i) 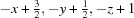.

**Table 3:** Experimental details

	(I)	(II)
Crystal data		
Chemical formula	C_14_H_2_F_8_N_2_O_4_·2C_2_H_6_O	C_14_H_2_F_8_N_2_O_4_
*M*_r_	506.31	414.18
Crystal system, space group	Triclinic, *P* 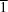	Monoclinic, *C*2/*c*
Temperature (K)	150	150
*a*, *b*, *c* (Å)	5.8188 (9), 10.4579 (16), 17.468 (3)	21.7297 (16), 6.5797 (5), 10.2247 (8)
α, β, γ (°)	99.186 (8), 99.112 (8), 99.950 (8)	90, 100.058 (4), 90
*V* (Å^3^)	1014.5 (3)	1439.41 (19)
*Z*	2	4
Radiation type	Ga *K*α, λ = 1.34139 Å	Ga *K*α, λ = 1.34139 Å
μ (mm^−1^)	0.98	1.21
Crystal size (mm)	0.25 × 0.08 × 0.03	0.15 × 0.08 × 0.04

Data collection		
Diffractometer	Bruker Venture Metaljet	Bruker Venture Metaljet
Absorption correction	Multi-scan (*SADABS*; Krause *et al.*, 2015 ▸)	Multi-scan (*SADABS*; Krause *et al.*, 2015 ▸)
*T*_min_, *T*_max_	0.549, 0.751	0.547, 0.752
No. of measured, independent and observed [*I* > 2σ(*I*)] reflections	18534, 18534, 13265	9647, 1656, 1339
*R*_int_	–	0.052
(sin θ/λ)_max_ (Å^−1^)	0.614	0.650

Refinement		
*R*[*F* ^2^ > 2σ(*F* ^2^)], *wR*(*F* ^2^), *S*	0.093, 0.288, 1.07	0.057, 0.171, 1.06
No. of reflections	18534	1656
No. of parameters	312	132
H-atom treatment	H-atom parameters constrained	All H-atom parameters refined
Δρ_max_, Δρ_min_ (e Å^−3^)	0.49, −0.41	0.25, −0.31

Computer programs: *APEX2* and *SAINT* (Bruker, 2013 ▸), *SHELXT* (Sheldrick, 2015*a* ▸), *SHELXL2018* (Sheldrick, 2015*b* ▸), *OLEX2* (Dolomanov *et al.*, 2009 ▸), *Mercury* (Macrae *et al.*, 2008 ▸) and *publCIF* (Westrip, 2010 ▸).

## supplementary crystallographic information

### cis-2,3,5,6,2',3',5',6'-Octafluoro-4,4'-azinodibenzoic acid ethanol disolvate (I) Crystal data

**Table:**

C_14_H_2_F_8_N_2_O_4_·2C_2_H_6_O	*Z* = 2
*M**_r_* = 506.31	*F*(000) = 512
Triclinic, *P*1	*D*_x_ = 1.658 Mg m^−^^3^
*a* = 5.8188 (9) Å	Ga *K*α radiation, λ = 1.34139 Å
*b* = 10.4579 (16) Å	Cell parameters from 9922 reflections
*c* = 17.468 (3) Å	θ = 2.3–55.1°
α = 99.186 (8)°	µ = 0.98 mm^−^^1^
β = 99.112 (8)°	*T* = 150 K
γ = 99.950 (8)°	Platelet, clear light yellow
*V* = 1014.5 (3) Å^3^	0.25 × 0.08 × 0.03 mm

### cis-2,3,5,6,2',3',5',6'-Octafluoro-4,4'-azinodibenzoic acid ethanol disolvate (I) Data collection

**Table:**

Bruker Venture Metaljet diffractometer	18534 measured reflections
Radiation source: Metal Jet, Gallium Liquid Metal Jet Source	18534 independent reflections
Helios MX Mirror Optics monochromator	13265 reflections with *I* > 2σ(*I*)
Detector resolution: 10.24 pixels mm^-1^	θ_max_ = 55.4°, θ_min_ = 2.3°
ω and φ scans	*h* = −7→7
Absorption correction: multi-scan (*SADABS*; Krause *et al*., 2015)	*k* = −12→12
*T*_min_ = 0.549, *T*_max_ = 0.751	*l* = −21→21

### cis-2,3,5,6,2',3',5',6'-Octafluoro-4,4'-azinodibenzoic acid ethanol disolvate (I) Refinement

**Table:**

Refinement on *F*^2^	Hydrogen site location: inferred from neighbouring sites
Least-squares matrix: full	H-atom parameters constrained
*R*[*F*^2^ > 2σ(*F*^2^)] = 0.093	*w* = 1/[σ^2^(*F*_o_^2^) + (0.1107*P*)^2^ + 2.0727*P*] where *P* = (*F*_o_^2^ + 2*F*_c_^2^)/3
*wR*(*F*^2^) = 0.288	(Δ/σ)_max_ < 0.001
*S* = 1.07	Δρ_max_ = 0.49 e Å^−^^3^
18534 reflections	Δρ_min_ = −0.41 e Å^−^^3^
312 parameters	Extinction correction: SHELXL-2018/3 (Sheldrick, 2015b), Fc^*^=kFc[1+0.001xFc^2^λ^3^/sin(2θ)]^-1/4^
0 restraints	Extinction coefficient: 0.012 (3)
Primary atom site location: dual	

### cis-2,3,5,6,2',3',5',6'-Octafluoro-4,4'-azinodibenzoic acid ethanol disolvate (I) Special details

**Table:**

Experimental. X-ray crystallographic data for I were collected from a single crystal sample, which was mounted on a loop fiber. Data were collected using a Bruker Venture diffractometer equipped with a Photon 100 CMOS Detector, a Helios MX optics and a Kappa goniometer. The crystal-to-detector distance was 4.0 cm, and the data collection was carried out in 1024 x 1024 pixel mode.
Geometry. All esds (except the esd in the dihedral angle between two l.s. planes) are estimated using the full covariance matrix. The cell esds are taken into account individually in the estimation of esds in distances, angles and torsion angles; correlations between esds in cell parameters are only used when they are defined by crystal symmetry. An approximate (isotropic) treatment of cell esds is used for estimating esds involving l.s. planes.
Refinement. Refined as a 2-component twin.

### cis-2,3,5,6,2',3',5',6'-Octafluoro-4,4'-azinodibenzoic acid ethanol disolvate (I) Fractional atomic coordinates and isotropic or equivalent isotropic displacement parameters (Å^2^)

**Table:**

	*x*	*y*	*z*	*U*_iso_*/*U*_eq_	
C1	0.9889 (11)	0.7393 (6)	0.2014 (3)	0.0597 (15)	
N1	1.1558 (10)	0.6593 (6)	0.2222 (3)	0.0672 (14)	
O1	0.3501 (7)	0.9771 (4)	0.1079 (2)	0.0653 (11)	
H1	0.280864	1.028028	0.084501	0.098*	
O2	0.6726 (7)	1.0908 (4)	0.0774 (2)	0.0618 (11)	
F2	1.2702 (6)	0.9263 (4)	0.2709 (2)	0.0770 (11)	
C2	1.0604 (10)	0.8764 (6)	0.2215 (3)	0.0584 (15)	
N2	1.1060 (10)	0.5642 (6)	0.2555 (3)	0.0687 (14)	
C3	0.9249 (11)	0.9584 (6)	0.1927 (3)	0.0595 (15)	
F3	1.0065 (7)	1.0886 (3)	0.2150 (2)	0.0708 (10)	
O3	0.2387 (10)	0.5170 (5)	0.4479 (3)	0.0861 (14)	
O4	0.2042 (10)	0.3099 (5)	0.3888 (3)	0.0897 (15)	
H4	0.083481	0.299282	0.409650	0.135*	
C4	0.7100 (10)	0.9097 (6)	0.1410 (3)	0.0546 (14)	
F5	0.4289 (6)	0.7189 (3)	0.0718 (2)	0.0708 (10)	
C5	0.6371 (10)	0.7738 (6)	0.1226 (3)	0.0563 (14)	
F6	0.6944 (7)	0.5586 (3)	0.1279 (2)	0.0705 (10)	
C6	0.7702 (11)	0.6899 (6)	0.1508 (3)	0.0589 (15)	
C7	0.5754 (11)	1.0025 (6)	0.1050 (3)	0.0565 (14)	
C8	0.8909 (11)	0.5411 (6)	0.2860 (3)	0.0605 (15)	
F9	0.9583 (7)	0.7550 (3)	0.3629 (2)	0.0771 (11)	
C9	0.8279 (12)	0.6306 (6)	0.3424 (3)	0.0617 (15)	
C10	0.6437 (12)	0.5963 (6)	0.3793 (3)	0.0618 (15)	
F10	0.5974 (8)	0.6915 (4)	0.4324 (2)	0.0811 (11)	
C11	0.5087 (11)	0.4687 (6)	0.3642 (3)	0.0590 (15)	
F12	0.4592 (7)	0.2513 (4)	0.2879 (2)	0.0802 (11)	
C12	0.5750 (12)	0.3767 (6)	0.3080 (3)	0.0636 (16)	
C13	0.7577 (12)	0.4126 (6)	0.2712 (3)	0.0626 (16)	
F13	0.8152 (8)	0.3206 (4)	0.2172 (2)	0.0811 (11)	
C14	0.3069 (12)	0.4362 (7)	0.4047 (4)	0.0656 (17)	
O6	−0.1599 (10)	0.2670 (5)	0.4551 (3)	0.0852 (14)	
H6	−0.212334	0.326189	0.481383	0.128*	
C17	−0.3034 (15)	0.1409 (7)	0.4531 (4)	0.084 (2)	
H17A	−0.472205	0.141429	0.433163	0.100*	
H17B	−0.289254	0.121575	0.507162	0.100*	
C18	−0.2239 (17)	0.0366 (8)	0.4004 (6)	0.104 (3)	
H18A	−0.222599	0.060904	0.348511	0.156*	
H18B	−0.333646	−0.048393	0.394220	0.156*	
H18C	−0.063561	0.028925	0.423832	0.156*	
O5	0.1322 (7)	1.1419 (4)	0.0494 (2)	0.0656 (11)	
H5	−0.008659	1.122255	0.055918	0.098*	
C15	0.2311 (12)	1.2757 (6)	0.0863 (4)	0.0688 (17)	
H15A	0.398341	1.297679	0.079716	0.083*	
H15B	0.230495	1.286243	0.143629	0.083*	
C16	0.0966 (13)	1.3697 (7)	0.0526 (4)	0.0741 (18)	
H16A	0.096171	1.359388	−0.004153	0.111*	
H16B	0.172803	1.460624	0.078785	0.111*	
H16C	−0.067309	1.350866	0.061155	0.111*	

### cis-2,3,5,6,2',3',5',6'-Octafluoro-4,4'-azinodibenzoic acid ethanol disolvate (I) Atomic displacement parameters (Å^2^)

**Table:**

	*U*^11^	*U*^22^	*U*^33^	*U*^12^	*U*^13^	*U*^23^
C1	0.063 (4)	0.066 (4)	0.050 (3)	0.020 (3)	0.007 (3)	0.009 (3)
N1	0.071 (3)	0.072 (4)	0.063 (3)	0.028 (3)	0.008 (3)	0.013 (3)
O1	0.058 (3)	0.069 (3)	0.073 (3)	0.017 (2)	0.013 (2)	0.018 (2)
O2	0.066 (3)	0.058 (3)	0.058 (2)	0.015 (2)	0.0071 (19)	0.0044 (19)
F2	0.066 (2)	0.079 (2)	0.075 (2)	0.0081 (18)	−0.0105 (18)	0.0125 (19)
C2	0.053 (3)	0.064 (4)	0.053 (3)	0.006 (3)	0.002 (3)	0.009 (3)
N2	0.078 (4)	0.070 (3)	0.061 (3)	0.027 (3)	0.010 (3)	0.010 (3)
C3	0.072 (4)	0.049 (3)	0.051 (3)	0.009 (3)	0.006 (3)	0.002 (3)
F3	0.079 (2)	0.057 (2)	0.066 (2)	0.0060 (17)	−0.0002 (17)	0.0014 (16)
O3	0.095 (4)	0.078 (3)	0.078 (3)	0.014 (3)	0.025 (3)	−0.011 (3)
O4	0.106 (4)	0.064 (3)	0.096 (3)	0.008 (3)	0.033 (3)	0.004 (3)
C4	0.057 (3)	0.060 (4)	0.044 (3)	0.012 (3)	0.009 (2)	0.006 (3)
F5	0.069 (2)	0.063 (2)	0.068 (2)	0.0076 (16)	−0.0095 (17)	0.0061 (17)
C5	0.056 (3)	0.062 (4)	0.043 (3)	0.008 (3)	0.002 (2)	−0.001 (3)
F6	0.087 (3)	0.055 (2)	0.062 (2)	0.0166 (17)	0.0014 (18)	0.0017 (16)
C6	0.072 (4)	0.051 (3)	0.049 (3)	0.012 (3)	0.004 (3)	0.002 (3)
C7	0.064 (4)	0.055 (4)	0.048 (3)	0.015 (3)	0.010 (3)	0.000 (3)
C8	0.072 (4)	0.064 (4)	0.048 (3)	0.026 (3)	0.008 (3)	0.010 (3)
F9	0.100 (3)	0.059 (2)	0.063 (2)	0.0061 (19)	0.0128 (19)	0.0005 (17)
C9	0.077 (4)	0.052 (4)	0.051 (3)	0.013 (3)	0.002 (3)	0.004 (3)
C10	0.076 (4)	0.059 (4)	0.047 (3)	0.022 (3)	0.003 (3)	0.001 (3)
F10	0.106 (3)	0.062 (2)	0.073 (2)	0.018 (2)	0.023 (2)	−0.0041 (18)
C11	0.069 (4)	0.060 (4)	0.045 (3)	0.019 (3)	0.000 (3)	0.004 (3)
F12	0.100 (3)	0.062 (2)	0.067 (2)	0.007 (2)	0.012 (2)	−0.0072 (17)
C12	0.079 (4)	0.052 (4)	0.050 (3)	0.015 (3)	−0.005 (3)	−0.001 (3)
C13	0.080 (4)	0.060 (4)	0.045 (3)	0.025 (3)	0.001 (3)	−0.001 (3)
F13	0.104 (3)	0.066 (2)	0.071 (2)	0.023 (2)	0.022 (2)	−0.0042 (18)
C14	0.074 (4)	0.068 (4)	0.049 (3)	0.016 (3)	−0.005 (3)	0.009 (3)
O6	0.098 (4)	0.061 (3)	0.095 (4)	0.017 (3)	0.022 (3)	0.007 (3)
C17	0.100 (6)	0.071 (5)	0.075 (4)	0.022 (4)	0.011 (4)	0.004 (4)
C18	0.111 (7)	0.074 (5)	0.119 (7)	0.022 (5)	0.023 (5)	−0.008 (5)
O5	0.063 (3)	0.061 (3)	0.070 (3)	0.0148 (19)	0.007 (2)	0.005 (2)
C15	0.065 (4)	0.068 (4)	0.068 (4)	0.009 (3)	0.004 (3)	0.007 (3)
C16	0.086 (5)	0.069 (4)	0.069 (4)	0.019 (4)	0.015 (4)	0.015 (3)

### cis-2,3,5,6,2',3',5',6'-Octafluoro-4,4'-azinodibenzoic acid ethanol disolvate (I) Geometric parameters (Å, º)

**Table:**

C1—N1	1.429 (8)	C10—F10	1.341 (7)
C1—C2	1.393 (8)	C10—C11	1.388 (9)
C1—C6	1.392 (8)	C11—C12	1.409 (9)
N1—N2	1.244 (7)	C11—C14	1.482 (9)
O1—H1	0.8400	F12—C12	1.331 (7)
O1—C7	1.303 (7)	C12—C13	1.356 (9)
O2—C7	1.206 (7)	C13—F13	1.359 (7)
F2—C2	1.345 (6)	O6—H6	0.8400
C2—C3	1.363 (8)	O6—C17	1.425 (9)
N2—C8	1.434 (8)	C17—H17A	0.9900
C3—F3	1.334 (6)	C17—H17B	0.9900
C3—C4	1.383 (8)	C17—C18	1.498 (11)
O3—C14	1.203 (8)	C18—H18A	0.9800
O4—H4	0.8400	C18—H18B	0.9800
O4—C14	1.318 (8)	C18—H18C	0.9800
C4—C5	1.380 (8)	O5—H5	0.8400
C4—C7	1.500 (8)	O5—C15	1.422 (7)
F5—C5	1.356 (6)	C15—H15A	0.9900
C5—C6	1.366 (8)	C15—H15B	0.9900
F6—C6	1.342 (7)	C15—C16	1.493 (9)
C8—C9	1.386 (8)	C16—H16A	0.9800
C8—C13	1.393 (9)	C16—H16B	0.9800
F9—C9	1.349 (7)	C16—H16C	0.9800
C9—C10	1.361 (9)		
			
C2—C1—N1	118.9 (5)	C12—C11—C14	123.9 (6)
C6—C1—N1	123.7 (6)	F12—C12—C11	121.5 (6)
C6—C1—C2	116.6 (5)	F12—C12—C13	117.1 (6)
N2—N1—C1	122.3 (5)	C13—C12—C11	121.5 (6)
C7—O1—H1	109.5	C12—C13—C8	122.8 (6)
F2—C2—C1	117.6 (5)	C12—C13—F13	119.5 (6)
F2—C2—C3	120.5 (5)	F13—C13—C8	117.8 (6)
C3—C2—C1	121.9 (5)	O3—C14—O4	122.1 (7)
N1—N2—C8	121.5 (5)	O3—C14—C11	123.8 (6)
C2—C3—C4	121.7 (5)	O4—C14—C11	114.2 (6)
F3—C3—C2	118.0 (5)	C17—O6—H6	109.5
F3—C3—C4	120.3 (5)	O6—C17—H17A	109.8
C14—O4—H4	109.5	O6—C17—H17B	109.8
C3—C4—C7	120.2 (5)	O6—C17—C18	109.5 (7)
C5—C4—C3	116.3 (5)	H17A—C17—H17B	108.2
C5—C4—C7	123.4 (5)	C18—C17—H17A	109.8
F5—C5—C4	119.5 (5)	C18—C17—H17B	109.8
F5—C5—C6	117.5 (5)	C17—C18—H18A	109.5
C6—C5—C4	122.9 (5)	C17—C18—H18B	109.5
C5—C6—C1	120.6 (5)	C17—C18—H18C	109.5
F6—C6—C1	119.3 (5)	H18A—C18—H18B	109.5
F6—C6—C5	120.1 (5)	H18A—C18—H18C	109.5
O1—C7—C4	113.0 (5)	H18B—C18—H18C	109.5
O2—C7—O1	125.3 (5)	C15—O5—H5	109.5
O2—C7—C4	121.7 (6)	O5—C15—H15A	109.2
C9—C8—N2	124.6 (6)	O5—C15—H15B	109.2
C9—C8—C13	115.6 (6)	O5—C15—C16	112.2 (5)
C13—C8—N2	118.5 (6)	H15A—C15—H15B	107.9
F9—C9—C8	118.7 (6)	C16—C15—H15A	109.2
F9—C9—C10	119.0 (6)	C16—C15—H15B	109.2
C10—C9—C8	122.3 (6)	C15—C16—H16A	109.5
C9—C10—C11	122.4 (6)	C15—C16—H16B	109.5
F10—C10—C9	117.1 (6)	C15—C16—H16C	109.5
F10—C10—C11	120.5 (6)	H16A—C16—H16B	109.5
C10—C11—C12	115.5 (6)	H16A—C16—H16C	109.5
C10—C11—C14	120.6 (6)	H16B—C16—H16C	109.5
			
C1—N1—N2—C8	−9.8 (9)	C5—C4—C7—O2	−130.9 (6)
C1—C2—C3—F3	180.0 (5)	C6—C1—N1—N2	−59.3 (8)
C1—C2—C3—C4	−0.9 (9)	C6—C1—C2—F2	179.4 (5)
N1—C1—C2—F2	−10.8 (8)	C6—C1—C2—C3	−0.7 (9)
N1—C1—C2—C3	169.2 (5)	C7—C4—C5—F5	−2.6 (8)
N1—C1—C6—C5	−168.7 (5)	C7—C4—C5—C6	174.7 (5)
N1—C1—C6—F6	9.2 (9)	C8—C9—C10—F10	−179.3 (5)
N1—N2—C8—C9	−58.6 (8)	C8—C9—C10—C11	1.3 (9)
N1—N2—C8—C13	135.3 (6)	F9—C9—C10—F10	2.5 (8)
F2—C2—C3—F3	−0.1 (8)	F9—C9—C10—C11	−176.8 (5)
F2—C2—C3—C4	179.1 (5)	C9—C8—C13—C12	1.3 (8)
C2—C1—N1—N2	131.6 (6)	C9—C8—C13—F13	−178.9 (5)
C2—C1—C6—C5	0.7 (9)	C9—C10—C11—C12	0.0 (8)
C2—C1—C6—F6	178.5 (5)	C9—C10—C11—C14	−178.9 (5)
C2—C3—C4—C5	2.3 (8)	C10—C11—C12—F12	−179.6 (5)
C2—C3—C4—C7	−174.8 (5)	C10—C11—C12—C13	−0.5 (8)
N2—C8—C9—F9	9.7 (8)	C10—C11—C14—O3	5.0 (9)
N2—C8—C9—C10	−168.4 (5)	C10—C11—C14—O4	−175.8 (5)
N2—C8—C13—C12	168.7 (5)	F10—C10—C11—C12	−179.4 (5)
N2—C8—C13—F13	−11.5 (8)	F10—C10—C11—C14	1.7 (8)
C3—C4—C5—F5	−179.6 (5)	C11—C12—C13—C8	−0.1 (9)
C3—C4—C5—C6	−2.3 (8)	C11—C12—C13—F13	−179.9 (5)
C3—C4—C7—O1	−133.7 (6)	F12—C12—C13—C8	179.0 (5)
C3—C4—C7—O2	45.9 (8)	F12—C12—C13—F13	−0.8 (8)
F3—C3—C4—C5	−178.6 (5)	C12—C11—C14—O3	−173.8 (6)
F3—C3—C4—C7	4.4 (8)	C12—C11—C14—O4	5.4 (8)
C4—C5—C6—C1	0.8 (9)	C13—C8—C9—F9	176.3 (5)
C4—C5—C6—F6	−177.0 (5)	C13—C8—C9—C10	−1.9 (8)
F5—C5—C6—C1	178.2 (5)	C14—C11—C12—F12	−0.8 (8)
F5—C5—C6—F6	0.4 (8)	C14—C11—C12—C13	178.3 (5)
C5—C4—C7—O1	49.5 (7)		

### cis-2,3,5,6,2',3',5',6'-Octafluoro-4,4'-azinodibenzoic acid ethanol disolvate (I) Hydrogen-bond geometry (Å, º)

**Table:**

*D*—H···*A*	*D*—H	H···*A*	*D*···*A*	*D*—H···*A*
O1—H1···O5	0.84	1.72	2.556 (5)	171.9
O4—H4···O6	0.84	1.74	2.579 (7)	175.7
O6—H6···O3^i^	0.84	1.93	2.751 (7)	164
O5—H5···O2^ii^	0.84	1.93	2.771 (6)	175.2

Symmetry codes: (i) −*x*, −*y*+1, −*z*+1; (ii) *x*−1, *y*, *z*.

### cis-2,3,5,6,2',3',5',6'-Octafluoro-4,4'-azinodibenzoic acid (II) Crystal data

**Table:**

C_14_H_2_F_8_N_2_O_4_	*F*(000) = 816
*M**_r_* = 414.18	*D*_x_ = 1.911 Mg m^−^^3^
Monoclinic, *C*2/*c*	Ga *K*α radiation, λ = 1.34139 Å
*a* = 21.7297 (16) Å	Cell parameters from 6326 reflections
*b* = 6.5797 (5) Å	θ = 3.6–60.6°
*c* = 10.2247 (8) Å	µ = 1.21 mm^−^^1^
β = 100.058 (4)°	*T* = 150 K
*V* = 1439.41 (19) Å^3^	Plate, clear light orange
*Z* = 4	0.15 × 0.08 × 0.04 mm

### cis-2,3,5,6,2',3',5',6'-Octafluoro-4,4'-azinodibenzoic acid (II) Data collection

**Table:**

Bruker Venture Metaljet diffractometer	1656 independent reflections
Radiation source: Metal Jet, Gallium Liquid Metal Jet Source	1339 reflections with *I* > 2σ(*I*)
Helios MX Mirror Optics monochromator	*R*_int_ = 0.052
Detector resolution: 10.24 pixels mm^-1^	θ_max_ = 60.7°, θ_min_ = 3.6°
ω and φ scans	*h* = −28→27
Absorption correction: multi-scan (*SADABS*; Krause *et al*., 2015)	*k* = −8→8
*T*_min_ = 0.547, *T*_max_ = 0.752	*l* = −13→13
9647 measured reflections	

### cis-2,3,5,6,2',3',5',6'-Octafluoro-4,4'-azinodibenzoic acid (II) Refinement

**Table:**

Refinement on *F*^2^	Hydrogen site location: difference Fourier map
Least-squares matrix: full	All H-atom parameters refined
*R*[*F*^2^ > 2σ(*F*^2^)] = 0.057	*w* = 1/[σ^2^(*F*_o_^2^) + (0.0917*P*)^2^ + 1.5671*P*] where *P* = (*F*_o_^2^ + 2*F*_c_^2^)/3
*wR*(*F*^2^) = 0.171	(Δ/σ)_max_ < 0.001
*S* = 1.06	Δρ_max_ = 0.25 e Å^−^^3^
1656 reflections	Δρ_min_ = −0.31 e Å^−^^3^
132 parameters	Extinction correction: SHELXL-2018/3 (Sheldrick, 2015b), Fc^*^=kFc[1+0.001xFc^2^λ^3^/sin(2θ)]^-1/4^
0 restraints	Extinction coefficient: 0.0040 (7)
Primary atom site location: dual	

### cis-2,3,5,6,2',3',5',6'-Octafluoro-4,4'-azinodibenzoic acid (II) Special details

**Table:**

Experimental. X-ray crystallographic data for I were collected from a single crystal sample, which was mounted on a loop fiber. Data were collected using a Bruker Venture diffractometer equipped with a Photon 100 CMOS Detector, a Helios MX optics and a Kappa goniometer. The crystal-to-detector distance was 4.0 cm, and the data collection was carried out in 1024 x 1024 pixel mode.
Geometry. All esds (except the esd in the dihedral angle between two l.s. planes) are estimated using the full covariance matrix. The cell esds are taken into account individually in the estimation of esds in distances, angles and torsion angles; correlations between esds in cell parameters are only used when they are defined by crystal symmetry. An approximate (isotropic) treatment of cell esds is used for estimating esds involving l.s. planes.

### cis-2,3,5,6,2',3',5',6'-Octafluoro-4,4'-azinodibenzoic acid (II) Fractional atomic coordinates and isotropic or equivalent isotropic displacement parameters (Å^2^)

**Table:**

	*x*	*y*	*z*	*U*_iso_*/*U*_eq_	
N1	0.52881 (9)	1.1272 (3)	0.2701 (2)	0.0493 (5)	
C1	0.56365 (10)	0.9427 (4)	0.3044 (2)	0.0451 (5)	
O1	0.74933 (8)	0.5029 (3)	0.46997 (18)	0.0550 (5)	
C2	0.62039 (10)	0.9234 (4)	0.2592 (2)	0.0466 (6)	
F2	0.63497 (7)	1.0570 (3)	0.17121 (15)	0.0598 (5)	
O2	0.66949 (9)	0.2838 (3)	0.4540 (2)	0.0601 (5)	
H2	0.699 (2)	0.185 (8)	0.473 (5)	0.142 (19)*	
F3	0.71428 (7)	0.7543 (2)	0.25407 (15)	0.0604 (5)	
C3	0.66112 (10)	0.7681 (4)	0.3031 (2)	0.0486 (6)	
C4	0.64750 (10)	0.6250 (4)	0.3932 (2)	0.0460 (6)	
C5	0.59134 (11)	0.6440 (4)	0.4392 (2)	0.0464 (5)	
F5	0.57659 (7)	0.5188 (2)	0.53098 (15)	0.0570 (5)	
C6	0.54993 (10)	0.8009 (4)	0.3945 (2)	0.0457 (5)	
F6	0.49705 (6)	0.8153 (2)	0.44498 (14)	0.0529 (4)	
C7	0.69243 (11)	0.4589 (4)	0.4432 (2)	0.0470 (6)	

### cis-2,3,5,6,2',3',5',6'-Octafluoro-4,4'-azinodibenzoic acid (II) Atomic displacement parameters (Å^2^)

**Table:**

	*U*^11^	*U*^22^	*U*^33^	*U*^12^	*U*^13^	*U*^23^
N1	0.0456 (10)	0.0508 (11)	0.0466 (10)	0.0015 (8)	−0.0054 (7)	0.0028 (8)
C1	0.0405 (11)	0.0486 (12)	0.0413 (11)	−0.0022 (9)	−0.0065 (8)	0.0003 (9)
O1	0.0393 (9)	0.0583 (11)	0.0618 (10)	0.0015 (7)	−0.0067 (7)	−0.0017 (8)
C2	0.0407 (11)	0.0533 (13)	0.0420 (11)	−0.0028 (9)	−0.0037 (8)	0.0051 (9)
F2	0.0521 (9)	0.0680 (10)	0.0569 (8)	0.0016 (7)	0.0033 (6)	0.0198 (7)
O2	0.0508 (10)	0.0501 (10)	0.0702 (12)	0.0000 (8)	−0.0153 (8)	0.0050 (8)
F3	0.0455 (8)	0.0788 (11)	0.0558 (9)	0.0081 (7)	0.0056 (6)	0.0135 (7)
C3	0.0393 (11)	0.0591 (14)	0.0437 (11)	−0.0006 (10)	−0.0033 (9)	0.0015 (10)
C4	0.0385 (11)	0.0486 (12)	0.0451 (12)	−0.0008 (9)	−0.0083 (8)	0.0011 (9)
C5	0.0454 (12)	0.0490 (13)	0.0408 (11)	−0.0039 (9)	−0.0039 (8)	0.0024 (9)
F5	0.0520 (8)	0.0581 (9)	0.0588 (9)	−0.0001 (6)	0.0035 (6)	0.0141 (7)
C6	0.0371 (11)	0.0526 (12)	0.0440 (11)	−0.0033 (9)	−0.0026 (8)	−0.0010 (9)
F6	0.0449 (8)	0.0576 (9)	0.0545 (8)	0.0010 (6)	0.0044 (6)	0.0041 (6)
C7	0.0429 (12)	0.0527 (13)	0.0412 (11)	0.0026 (9)	−0.0048 (8)	−0.0014 (9)

### cis-2,3,5,6,2',3',5',6'-Octafluoro-4,4'-azinodibenzoic acid (II) Geometric parameters (Å, º)

**Table:**

N1—N1^i^	1.248 (4)	O2—C7	1.268 (3)
N1—C1	1.441 (3)	F3—C3	1.340 (3)
C1—C2	1.396 (3)	C3—C4	1.385 (3)
C1—C6	1.380 (3)	C4—C5	1.388 (3)
O1—C7	1.253 (3)	C4—C7	1.495 (3)
C2—F2	1.335 (3)	C5—F5	1.329 (3)
C2—C3	1.374 (3)	C5—C6	1.393 (3)
O2—H2	0.90 (5)	C6—F6	1.343 (3)
			
N1^i^—N1—C1	122.30 (12)	C3—C4—C7	121.4 (2)
C2—C1—N1	117.0 (2)	C5—C4—C7	120.7 (2)
C6—C1—N1	124.5 (2)	C4—C5—C6	120.8 (2)
C6—C1—C2	117.7 (2)	F5—C5—C4	121.2 (2)
F2—C2—C1	119.3 (2)	F5—C5—C6	118.0 (2)
F2—C2—C3	119.6 (2)	C1—C6—C5	121.2 (2)
C3—C2—C1	121.0 (2)	F6—C6—C1	120.5 (2)
C7—O2—H2	114 (3)	F6—C6—C5	118.2 (2)
C2—C3—C4	121.5 (2)	O1—C7—O2	125.4 (2)
F3—C3—C2	118.4 (2)	O1—C7—C4	117.8 (2)
F3—C3—C4	120.1 (2)	O2—C7—C4	116.8 (2)
C3—C4—C5	117.8 (2)		
			
N1^i^—N1—C1—C2	136.0 (3)	F3—C3—C4—C7	3.1 (3)
N1^i^—N1—C1—C6	−54.6 (4)	C3—C4—C5—F5	−176.40 (19)
N1—C1—C2—F2	−10.4 (3)	C3—C4—C5—C6	0.8 (3)
N1—C1—C2—C3	170.2 (2)	C3—C4—C7—O1	40.6 (3)
N1—C1—C6—C5	−169.2 (2)	C3—C4—C7—O2	−138.9 (2)
N1—C1—C6—F6	8.4 (3)	C4—C5—C6—C1	−0.6 (3)
C1—C2—C3—F3	178.5 (2)	C4—C5—C6—F6	−178.21 (19)
C1—C2—C3—C4	0.2 (4)	C5—C4—C7—O1	−137.4 (2)
C2—C1—C6—C5	0.1 (3)	C5—C4—C7—O2	43.1 (3)
C2—C1—C6—F6	177.73 (19)	F5—C5—C6—C1	176.7 (2)
C2—C3—C4—C5	−0.6 (3)	F5—C5—C6—F6	−1.0 (3)
C2—C3—C4—C7	−178.6 (2)	C6—C1—C2—F2	179.49 (19)
F2—C2—C3—F3	−1.0 (3)	C6—C1—C2—C3	0.1 (3)
F2—C2—C3—C4	−179.3 (2)	C7—C4—C5—F5	1.7 (3)
F3—C3—C4—C5	−178.8 (2)	C7—C4—C5—C6	178.8 (2)

Symmetry code: (i) −*x*+1, *y*, −*z*+1/2.

### cis-2,3,5,6,2',3',5',6'-Octafluoro-4,4'-azinodibenzoic acid (II) Hydrogen-bond geometry (Å, º)

**Table:**

*D*—H···*A*	*D*—H	H···*A*	*D*···*A*	*D*—H···*A*
O2—H2···O1^ii^	0.90 (5)	1.71 (5)	2.607 (2)	173 (5)

Symmetry code: (ii) −*x*+3/2, −*y*+1/2, −*z*+1.
